# Age-stratified prevalence of latent tuberculosis infection in Ulaanbaatar, Mongolia: a facility-based cross-sectional study

**DOI:** 10.1016/j.ijregi.2026.100944

**Published:** 2026-07-07

**Authors:** Erdenegerel Narmandakh, Enkhtsetseg Tumur, Gundsuren Sharkhuu, Yuriko Igarashi, Gantungalag Ganbaatar, Naranzul Dambaa, Nyamjargal Batbayar, Gantsetseg Dorj, Tsetsegtuya Borolzoi, Odmaa Enkhtaivan, Munkhzul Baatar, Enkhbolor Davaakhuu, Bilegtsaikhan Tsolmon, Sarantuya Jav, Buyankhishig Burneebaatar, Satoshi Mitarai

**Affiliations:** 1 National Center for Communicable Diseases, Ulaanbaatar, Mongolia; 2 Mongolian National University of Medical Sciences, Ulaanbaatar, Mongolia; 3 Research Institute of Tuberculosis, Japan Anti-Tuberculosis Association, Tokyo, Japan

**Keywords:** Latent tuberculosis infection, QuantiFERON-TB Gold Plus, Age-stratified, Mongolia

## Abstract

**Objectives:**

Mongolia has a high tuberculosis (TB) burden. This study aimed to assess the age-specific latent TB infection (LTBI) prevalence in a healthy population in Mongolia by using interferon-gamma release assays.

**Methods:**

This facility-based cross-sectional study was conducted from June 2024 to March 2025 in Ulaanbaatar. Eligible participants were asymptomatic, aged ≥10 years, had no previous TB treatment, and had no known contact with TB. LTBI was detected using the QuantiFERON-TB Gold Plus (QFT-Plus) assay. Sociodemographic data were collected using standardized questionnaires.

**Results:**

The overall QFT-Plus positivity rate in 766 eligible participants (mean age: 38.2 years; 47.1% female) was 33.4%. Age-specific positivity increased from 7.9% (participants aged 10-19 years) to 46.1% (participants aged ≥60 years, *P* < 0.0001). Sex was not associated with positivity (*P* = 0.652), although the rate was slightly higher in women aged 40-59 years than in men. Multivariable analysis confirmed that age was a significant predictor of QFT-Plus positivity, reflecting TB prevalence.

**Conclusion:**

Ulaanbaatar’s LTBI prevalence remains high, especially among older adults, suggesting a shift from recent transmission to remote infection reactivation. Although QFT-Plus positivity in young individuals remains high, our findings support the prioritization of targeted preventive strategies for older populations.

## Highlights

• Age was significantly associated with higher tuberculosis (TB) infection rates in Ulaanbaatar.
• Sex was not a determining factor for TB infection in Ulaanbaatar, Mongolia.
• There was an age-wise increase in latent TB infection in Ulaanbaatar, Mongolia.
• Overall QFT-Plus positivity among 766 participants was 33.4%.

## Introduction

Tuberculosis (TB) is caused by the bacillus *Mycobacterium tuberculosis* and is transmitted via the airborne route when people with active TB cough, sneeze, or speak. In 2023, the World Health Organization (WHO) estimated that 10.8 million people developed TB and 1.25 million died of the disease globally [1]. An estimated 1.7 billion people (23% of the world’s population) are latently infected with TB, and active TB cases arise from these latent infections [1]. Therefore, the detection of TB and preventive treatment are essential for the global End TB Strategy.

Mongolia was among the countries with a high TB burden in 2021, and the TB incidence rate in 2023 was 491 per 100,000 population, which was 3.7-5.1 times higher than the average globally and in Western Pacific countries [1]. In 2023, 3181 cases of TB were reported in Mongolia, representing a marginal decline of 0.5% compared with the previous year. Of these, 2357 (74%) were newly diagnosed, corresponding to a national notification rate of 68 per 100,000 population. Notably, 1344 (57.0%) of the new cases were registered in Ulaanbaatar, where the notification rate (79 cases per 100,000 population) exceeded the national average, underscoring the capital as a key area of TB transmission. Although the absolute number of new cases was highest among individuals aged 15-44 years, the notification rate was comparatively higher among those aged 45 years and older, indicating that age is a significant factor in the epidemiology of TB [2].

According to the Mongolian Statistical Information Service, Ulaanbaatar accounted for approximately 49.5% of the national population as of 2023. Significantly, approximately 57% of all TB cases in the country are reported in Ulaanbaatar, indicating a substantial concentration of the disease burden in the city [2]. Given this context, Ulaanbaatar should be prioritized for intensified investments in TB control measures, including the deployment of more effective screening tools, to help reduce the reservoir of latent and active TB infections. This study aimed to assess the prevalence of TB infection in a relatively healthy population stratified by age group in a densely populated urban setting with a high TB incidence in Ulaanbaatar.

## Methods

### Study setting, design, and population

This facility-based cross-sectional study was conducted between 13 June 2024 and 21 March 2025 at the National Center for Communicable Diseases in Ulaanbaatar, Mongolia, focusing on TB infection rates among healthy individuals from an early screening program of the government, routine health checkups of company employees, and general screening in secondary schools. Participants were categorized by age into groups aged 10-19, 20-29, 30-39, 40-49, 50-59, and ≥60 years, with a target of at least 100 individuals per age group and a minimum overall sample of 600. This study investigated the correlations between age, sex, sociodemographic variables, and QuantiFERON-TB Gold Plus (QFT-Plus; Qiagen Diagnostics, Hilden, Germany) results.

The participants completed a questionnaire containing questions on sociodemographic factors, TB symptoms, and contacts. After completing the questionnaire, participants recruited through the government early TB detection screening program underwent chest radiography (219/766, 28.6%), followed by testing with the QFT-Plus assay using whole blood samples for all participants. For participants recruited through routine health checkups of company employees and general screening in secondary schools, active TB was excluded based on symptom questionnaires, clinical examination, and epidemiological history rather than chest radiography. This design provided an overview of the population's TB infection status and related risk factors at a particular time.

### Inclusion criteria

Eligible individuals were healthy and asymptomatic, aged ≥10 years, had not been previously treated for TB, and had no known contact with TB.

The study excluded participants with signs and symptoms of active TB, confirmed cases, or close contact with patients with active pulmonary TB as identified by the study questionnaire.

### Interferon-gamma release assay

5-6 mL of whole blood containing lithium heparin was used for the QFT-Plus. Blood samples were processed according to the guidelines provided in the manufacturer’s QFT-Plus package insert. Interferon-gamma was determined using an enzyme-linked immunosorbent assay according to the manufacturer’s instructions. The optical densities generated from the automated Multiskan SkyHigh enzyme-linked immunosorbent assay microplate reader (Thermo Fisher Scientific) were then transferred to the QFT-Plus analysis software to obtain quantitative values. Quantitative values were reported in international units (IUs) per mL (IU/mL). The Nil tube provided the background concentration; hence, all QFT-Plus responses were evaluated by subtracting the Nil response. A cutoff value of 0.35 IU/mL was used.

### Data management and statistical analysis

Python (version 3.8.8) and pandas (1.2.4), NumPy (1.20.3), statsmodels (0.12.2), and seaborn (0.11.1) libraries were used to analyze the demographic information and QFT-Plus results. Statistical tests were used to compare the differences between the positive and negative groups for each continuous variable, including the F-test (analysis of variance), which was used to compare the mean differences between the positive and negative groups of each continuous variable, and to determine whether the differences were significant. The chi-square test was performed to test the relationship between the variables and the QFT-Plus results for categorical variables. The 95% confidence interval (CI) of the QFT-Plus positivity rate was calculated for each age group and sex using the Wald method, which is a range of values for the proportion of positive cases. The prevalence of positive QFT-Plus results and weighted prevalence of latent TB infection (LTBI) were calculated considering the proportion of each age group in the population. A chi-square test was performed to assess the independence of sex, age group, and QFT-Plus positivity. The 95% CIs for the proportion of positive QFT-Plus results were also calculated for each age group and sex.

### Ethical approval

All participants provided written informed consent confirming their voluntary participation and understanding of the study. This study was approved by the Medical Ethics Review Committee of the Ministry of Health in Mongolia (reference number 24/048, issued on 27 May 2024).

## Results

**Figure 1:**
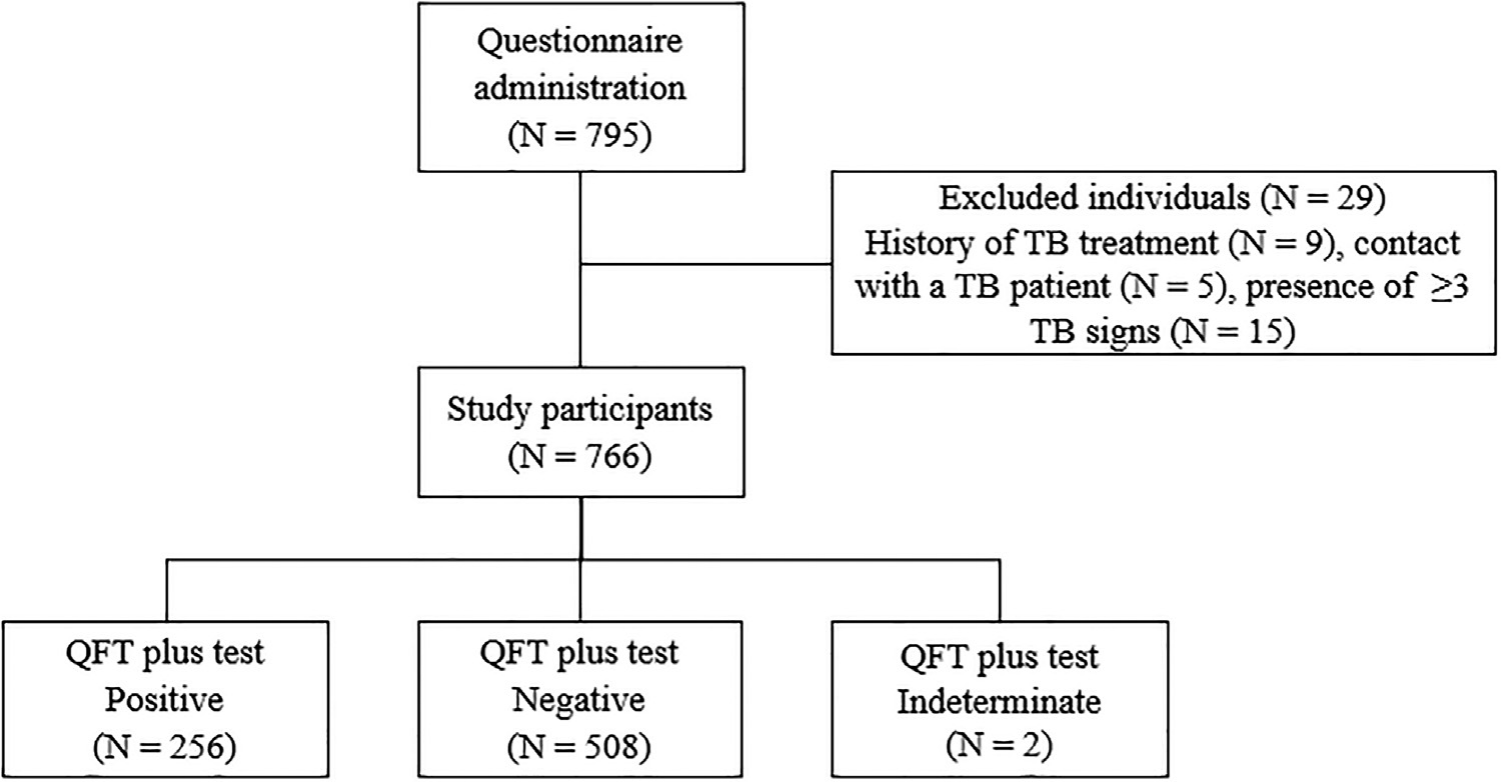
Flowchart of the participants in this study.

In total, 795 individuals participated in the survey (Figure 1). After excluding 29 participants with a history of TB treatment, known contact with patients with TB, or signs and symptoms of active TB, 766 participants were included in the final analysis. Two participants (0.3%) had indeterminate QFT-Plus results because of a low mitogen response and a high Nil response. The overall QFT-Plus positivity rate in the tested population was 33.4%.

**Figure 2:**
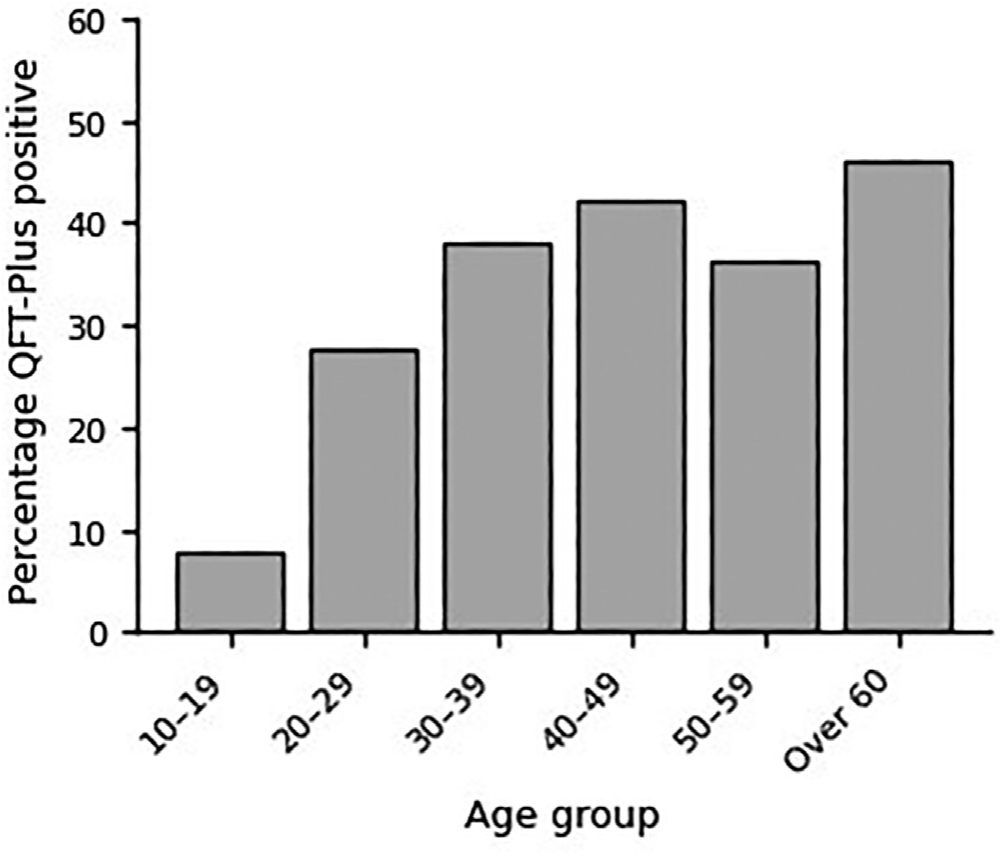
QFT-Plus results and positivity rate by age group. Age-specific percentage of positive interferon-gamma release assay (IGRA) results. Bars represent the percentage of individuals with a positive QFT-Plus result within each age group. A significant increasing trend with age was observed (Cochran–Armitage test for trend, *P* < 0.0001).

Abbreviations: IGRA, interferon-gamma release assay; QFT-Plus, QuantiFERON-TB Gold Plus.

After adjusting for age distribution through weighted prevalence estimation, the figure marginally increased to 33.5%, indicating that approximately one-third of the study population may have TB infection. Table 1 presents the characteristics of all 766 participants. Among the six age groups, participants aged 30-39 years constituted the largest proportion (26.5%), whereas those aged ≥60 years constituted the smallest proportion (11.6%). The mean number of family members was 3.7 (SD, 1.51). Figure 2 shows that the QFT-Plus positivity rate increased with age and was lowest in young adults and highest in those aged ≥60 years.

**Table 1:** Characteristics of the study participants.

Variables	N	Percentage
Sex		
Female	361	47.1%
Male	405	52.9%
Age groups		
10-19 years	91	11.9%
20-29 years	167	21.8%
30-39 years	203	26.5%
40-49 years	114	14.9%
50-59 years	102	13.3%
≥60 years	89	11.6%
Marital status		
Married	480	62.7%
Not married	286	37.3%
Education		
No formal education	-	-
Primary school	6	0.8%
Lower secondary school	100	13.1%
Upper secondary school	328	42.8%
Technical	34	4.4%
Bachelor's degree	298	38.9%
Employment status		
Unemployed	215	28.1%
Employed	551	71.9%
IGRA results		
Negative	508	66.3%
Positive	256	33.4%
Indeterminate	2	0.3%
Average number of family members	3.7	

Abbreviation: IGRA, interferon-gamma release assay.

To investigate the associations between categorical variables and QFT-Plus test results, a series of independent chi-square tests were conducted. The analysis revealed that age group was significantly associated with QFT-Plus positivity (χ² = 48.3522, *P* < 0.0001), indicating a strong relationship between age and TB infection status. Marital status similarly demonstrated a highly significant association with QFT-Plus positivity (χ² = 15.6985, *P* = 0.0004) (Table 2).

**Table 2:** Association between sociodemographic variables and QFT-Plus test outcomes.

Sociodemographic variables	Chi-squared test	*P*-value
Sex	0.3170	0.8534
Age	157.0559	0.1276
Age group	48.3522	0.0000
Marital status	15.6985	0.0004
Education	9.8599	0.0138
Employment status	9.5927	0.0083
Family members	30.9061	0.0138

QFT-Plus, QuantiFERON-TB Gold Plus.

Other sociodemographic variables, including level of education (*P* = 0.0033), number of family members residing in the household (χ² = 30.9061, *P* = 0.0138), and employment status (χ² = 9.5927, *P* = 0.0083), also showed significant relationships with QFT-Plus positivity. These findings suggest that while these factors may not be as influential as age or marital status, they nonetheless contribute meaningfully to the variation in TB infection. In contrast, participant sex (χ² = 0.2307, *P* = 0.6310) was not significantly associated with positivity.

Analysis of variance was performed to assess the differences in test results across groups. The results indicated that age exerted a highly significant effect on QFT-Plus outcomes (F = 28.1358, *P* < 0.0001), reinforcing earlier findings from the chi-square test and *t*-test. Marital status also had a significant effect (F = 12.5253, *P* = 0.0004), suggesting that these categorical variables contributed substantially to the observed variance in QFT-Plus results.

To further model the likelihood of QFT-Plus positivity based on demographic factors, a logistic regression analysis was conducted with age group and sex as predictor variables. The intercept of the model was −2.5036, representing the baseline log-odds of testing positive when all covariates were at the reference levels. Compared with the reference age group (10-19 years), the following age groups exhibited significantly increased odds of QFT-Plus positivity: 20-29 years (coefficient = 1.4905, *P* = 0.001), 30-39 years (coefficient = 1.9655, *P* < 0.0001), 40-49 years (coefficient = 2.1420, *P* < 0.0001), 50-59 years (coefficient = 1.9127, *P* < 0.0001), and ≥60 years (coefficient = 2.3403, *P* < 0.0001). These findings indicated a progressive increase in the probability of a positive QFT-Plus result with age. In contrast, sex was not a significant predictor of test positivity (coefficient = 0.0733, *P* = 0.652), consistent with earlier findings, thus reaffirming the lack of a significant association between sex and TB infection status in this cohort (Table 3).

**Table 3:** Age group and sex distribution of the study participants.

Age group	Sex	N	QFT-Pluspositive result	Positivity rate (%)	95% CI (%)
10-19 years	Male	53	4	7.55%	0.44-14.66
	Female	38	3	7.89%	−0.68 to 16.47
20-29 years	Male	63	17	26.98%	16.02-37.94
	Female	104	29	27.88%	19.27-36.50
30-39 years	Male	77	32	41.56%	30.55-52.57
	Female	126	45	35.71%	27.35-44.08
40-49 years	Male	47	17	36.17%	22.43-49.91
	Female	67	31	46.27%	34.33-58.21
50-59 years	Male	64	20	31.25%	19.89-42.61
	Female	38	17	44.74%	28.93-60.55
Over 60 years	Male	57	27	47.37%	34.41-60.33
	Female	32	14	43.75%	26.56-60.94

Abbreviations: CI, confidence interval; QFT-Plus, QuantiFERON-TB Gold Plus.

## Discussion

In this study, we investigated the age-specific prevalence of LTBI in a healthy population in Ulaanbaatar, Mongolia, using interferon-gamma release assays (IGRAs). We found that age-specific positivity increased with age and that sex was not associated with positivity, reflecting the TB prevalence in Mongolia.

A meta-analysis of 15 studies in 2024 showed that the prevalence of LTBI in Asia was 21% (95% CI: 19%-23%) and 36% (95% CI: 12%-59%) according to the IGRAs and tuberculin skin test (TST) (cutoff: 10 mm) results, respectively [3]. Mongolia conducted nationwide TB screening between 1959 and 1960 and examined 815,988 individuals, accounting for 88% of the population at the time. This large-scale screening using TSTs revealed that 33% of the population had LTBI, while 1.48% were actively affected by the disease, marking the first comprehensive national estimate of TB prevalence. In Ulaanbaatar, the TB infection rate among the general population was reported to be 44.6%. TB infection rates varied significantly across provinces, ranging from <20% in some areas to nearly 60% in others [4]. Mongolia's Ministry of Health launched a national campaign titled “Prevention, early detection, and diagnosis of communicable and non-communicable diseases based on age, gender, and population health risk” from May 2022 to May 2024. Of the 869,624 persons who participated in the nationwide early TB detection screening, 11,479 (1.3%) were considered to have presumptive TB based on questionnaires. Of these, 805 individuals underwent testing to confirm a TB diagnosis, and eventually, 40 tested positive for it [5].

Recent studies have highlighted the age-related distribution of TB infections. In 2017, Gurjav et al. examined TB infections among 280 household contacts of patients with TB in Mongolia. They used both TST and QFT-Plus and found a high prevalence of LTBI, with 57.1% testing positive using the TST and 46.8% using the IGRA. The agreement between the tests was fair (κ = 0.374), and infection was more common among individuals older than 15 years. Additionally, CD8+ T-cell responses, suggestive of recent transmission, were detected in 45.6% of the QFT-Plus-positive individuals [6].

Similarly, Ganmaa et al. [7] conducted a cross-sectional study between 2015 and 2017 to identify risk factors associated with active TB among children aged 6 to 13 years who tested positive using the QuantiFERON®-TB Gold assay. The study was conducted across 18 schools in Ulaanbaatar, Mongolia, and included 9814 children, of whom 938 (9.6%) were positive.

In our study, the positivity rate among individuals aged 10-19 years was 7.9% (95% CI: 2.3%-13.6%), which is lower than the rate reported by Ganmaa et al. [7]. In a separate study, Ganmaa et al. [8] examined LTBI, vitamin D status, and lifestyle risk factors among healthcare workers in Ulaanbaatar, Mongolia. Data from 200 female and 41 male healthcare workers aged 22-61 years showed a QFT-Plus positivity rate of 47%. Age was a significant predictor of QFT-Plus positivity even after adjusting for sex, Prime Diet Quality Score, Pittsburgh Sleep Quality Index, and weekly physical activity levels. Specifically, each additional year of age was associated with an odds ratio (OR) of 1.054 (95% CI 1.024-1.087; *P* < 0.05) [8].

Our study also revealed that age was significantly associated with QFT-Plus positivity (χ² = 48.3522, *P* < 0.0001), indicating a strong relationship between age and TB infection status. International studies also reinforce the association between LTBI and age. Korean studies found that IGRA positivity increased significantly with age, with a slope of 0.0929 (*P* < 0.001) [9] and age was the single most important risk factor for LTBI and outweighed the effects of other variables [10]. A population-based, multicenter prospective cohort study by Gao et al. [11] covering 21,022 participants suggested outcomes similar to those of our study. The TB infection rate was low in participants younger than 20 years of age and gradually increased with age [11].

Global TB control strategies have set a target of treating 30 million individuals with LTBI. Considering that ending the TB epidemic is unattainable without robust prevention efforts, there is a need to prioritize preventive treatments [12]. Populations at the highest risk of progression from latent to active TB include household contacts of individuals with pulmonary TB and individuals with silicosis, which is caused by occupational exposure to silica dust [13]. Similarly, in a 2021 study of 1856 adults aged 65 years and older in Zhejiang Province of China, 34.9% were found to have LTBI and 0.5% developed active TB during a 1-year follow-up. A higher prevalence of LTBI was observed in individuals who frequented enclosed recreational spaces (39.5%). Multivariable analysis identified male sex (OR: 1.32), smoking (OR: 1.43), infrequent home ventilation (OR: 1.88), and abnormal chest radiography findings (OR: 1.48) as significant risk factors for LTBI [14]. In our study, although the rate was slightly higher in women aged 40-59 years than in men, sex was not associated with QFT-Plus positivity (χ² = 0.2307, *P* = 0.6310), which was consistent with the results of some studies [[15], [16], [17]].

Mongolia is taking decisive steps to combat TB. Aligned with the WHO’s End TB Strategy and Sustainable Development Goals, the country is finalizing its National Strategic Plan for TB 2025-2028 to accelerate its goal of ending TB. The plan set an ambitious target to reduce the incidence of TB from 491 per 100,000 in 2023 to 197 per 100,000 by 2028 [18]. Mongolia’s health system optimization approach, guided by the WHO, is a key pillar in strengthening its health system, particularly primary health care, to improve TB prevention and care. Aligned with the global priorities set by the WHO, this approach involves a major policy and operational shift toward integrating TB services into primary healthcare, making care more accessible, increasing TB case notifications, improving patient outcomes, and reducing the burden on specialized TB facilities [19,20].

This study had several limitations. First, because of insufficient chest radiographic coverage, active TB could not be fully excluded, and the chest radiography results were therefore omitted from the data analysis. Among 766 participants, the overall QFT-Plus positivity rate was 33.4% (256/766). QFT-Plus positivity among participants with X-ray available was 39.7% (87/219), compared with 30.9% (169/547) among those without X-ray availability. Although this difference was statistically significant (*P* = 0.024), the magnitude was modest, and positivity rates in both groups were close to the overall rate, suggesting that X-ray availability had limited practical impact on overall QFT positivity in this population. Additionally, sputum-based bacteriological testing and imaging for extrapulmonary TB were not performed, as the study protocol focused on characterizing LTBI status rather than diagnosing active or extrapulmonary TB; therefore, the possibility of undetected active or extrapulmonary TB cannot be completely excluded. Although our study was not randomized, the observed patterns highlight the importance of addressing age-specific risks and environmental factors for TB prevention. As Ulaanbaatar is the most densely populated city in Mongolia and accounts for a disproportionately high share of the national TB burden, the elevated QFT-Plus positivity observed in this study may partly reflect population density-related transmission dynamics specific to this urban setting. Since all participants resided in Ulaanbaatar, the generalizability of these findings to less densely populated provinces remains uncertain, and comparative studies across regions with varying population density are warranted to further clarify this relationship. Despite these limitations, this study showed that the prevalence of TB infection remains high.

## Conclusion

Age was significantly associated with higher TB infection rates, whereas sex was not a determining factor. In previous studies, most patients with LTBI were younger. However, the current study showed an increase with age in LTBI in the Mongolian setting, indicating a decrease in active transmission in young individuals and a shift to a situation in which TB from LTBI is predominant. This study emphasizes the shift in the TB infection situation in Mongolia from active transmission among young people to age-related active TB from LTBI and may be the first evidence of the TB situation in the country. The study findings are expected to inform and strengthen targeted strategies and serve as an evidence base for the age distribution of TB infections in Ulaanbaatar, Mongolia.

## CRediT authorship contribution statement

**Erdenegerel Narmandakh:** Data curation, Investigation, Writing – original draft, Writing – review & editing. **Enkhtsetseg Tumur:** Investigation, Writing – review & editing. **Gundsuren Sharkhuu:** Investigation, Writing – review & editing. **Yuriko Igarashi:** Methodology, Writing – review & editing. **Gantungalag Ganbaatar:** Project administration. **Naranzul Dambaa:** Formal analysis, Writing – original draft, Writing – review & editing. **Nyamjargal Batbayar:** Data curation, Writing – original draft, Writing – review & editing. **Gantsetseg Dorj:** Data curation, Writing – review & editing. **Tsetsegtuya Borolzoi:** Investigation, Project administration, Writing – review & editing. **Odmaa Enkhtaivan:** Project administration, Writing – review & editing. **Munkhzul Baatar:** Data curation, Writing – review & editing. **Enkhbolor Davaakhuu:** Investigation, Writing – review & editing. **Bilegtsaikhan Tsolmon:** Project administration, Writing – review & editing. **Sarantuya Jav:** Methodology, Writing – review & editing. **Buyankhishig Burneebaatar:** Funding acquisition, Methodology, Project administration, Writing – original draft, Writing – review & editing. **Satoshi Mitarai:** Conceptualization, Funding acquisition, Methodology, Project administration, Supervision, Writing – review & editing.

## Declaration of competing interest

The authors have no competing interests to declare.
